# Risk of violence and frailty syndrome among older adults treated at a hospital service

**DOI:** 10.1590/0034-7167-2022-0278

**Published:** 2023-05-29

**Authors:** Jefferson da Silva Soares, Ana Carolina dos Santos, Renata Clemente dos Santos-Rodrigues, Gleicy Karine Nascimento de Araújo-Monteiro, Barbara Maria Lopes da Silva Brandão, Rafaella Queiroga Souto

**Affiliations:** I Universidade Federal da Paraíba. João Pessoa, Paraíba, Brazil

**Keywords:** Elder Abuse, Violence, Aged, Geriatric Nursing, Frailty., Abuso de Ancianos, Violencia, Anciano, Enfermería Geriátrica, Fragilidad., Abuso de Idosos, Violência, Idoso, Enfermagem Geriátrica, Fragilidade.

## Abstract

**Objectives:**

to assess the association between risk of violence and frailty syndrome among hospitalized older adults.

**Methods:**

quantitative, analytical and cross-sectional research, carried out with older adults in two university hospitals. Data collection was performed using the Brazil Old Age Schedule, Hwalek-Sengstock Elder Abuse Screening Test and Edmonton Frail Scale instruments. It was analyzed using descriptive statistics and inferential statistics.

**Results:**

risk of violence was higher among women (68.9%), over 70 years old (64.7%), with more than 3 years of study (68.9%), without relationship (67.1%), who do not work (65.1%) and with income above 1 minimum wage (65.2%). There is a significant association between risk of violence and frailty (72.3%; p<0.001) and a positive correlation between the instrument scores (r=0.350; p-value<0.001).

**Conclusions:**

risk of violence was associated with being female and frailty. The study is expected to encourage further discussions related to the theme and nursing practice.

## RESUMO

**Objetivos:**

avaliar a associação entre o risco de violência e a síndrome da fragilidade entre idosos hospitalizados.

**Métodos:**

pesquisa quantitativa, analítica e transversal, realizada com idosos em dois hospitais universitários. A coleta de dados foi realizada através dos instrumentos *Brazil Old Age Schedule, Hwalek-Sengstock Elder Abuse Screening Test* e *Edmonton Frail Scale*. Analisou-se através de estatística descritiva e estatística inferencial.

**Resultados:**

o risco de violência foi maior entre mulheres (68,9%), acima de 70 anos (64,7%), com mais de 3 anos de estudo (68,9%), sem relacionamento (67,1%), que não trabalham (65,1%) e com renda superior a 1 salário mínimo (65,2%). Existe associação significativa entre o risco de violência e fragilidade (72,3%; p<0,001) e correlação positiva entre os escores dos instrumentos (r=0,350; p-valor<0,001).

**Conclusões:**

o risco de violência se associou com o gênero feminino e a fragilidade. Espera-se que o estudo fomente maiores discussões relacionadas à temática e à prática da enfermagem.

## RESUMEN

**Objetivos:**

evaluar la asociación entre el riesgo de violencia y el síndrome de fragilidad entre ancianos hospitalizados.

**Métodos:**

investigación cuantitativa, analítica y transversal, realizada con ancianos en dos hospitales universitarios. La recolección de datos se realizó utilizando los instrumentos Brazil Old Age Schedule, Hwalek-Sengstock Elder Abuse Screening Test y Edmonton Frail Scale. Se analizó mediante estadística descriptiva y estadística inferencial.

**Resultados:**

el riesgo de violencia fue mayor entre las mujeres (68,9%), mayores de 70 años (64,7%), con más de 3 años de estudio (68,9%), sin relación (67,1%), que no trabajan (65,1%) y con renta superior a 1 salario mínimo (65,2%). Existe asociación significativa entre riesgo de violencia y fragilidad (72,3%; p<0,001) y correlación positiva entre los puntajes del instrumento (r=0,350; p-valor<0,001).

**Conclusiones:**

el riesgo de violencia se asoció con el género femenino y la fragilidad. Se espera que el estudio fomente más discusiones relacionadas con el tema y la práctica de enfermería.

## INTRODUCTION

The world population continues at an increasing pace of aging, and the perspective is that the elderly population will reach the number of 2 billion in 2050. It is estimated that, nowadays, more than 125 million people are 80 years or older. These changes impact the socio-demographic situation and demand adaptations from the State and society^(1)^.

Aging can be considered a process that depends on the biochemical and social influences of each individual and, sometimes, are a consequence of the lifestyle adopted throughout life, social relationships and access to education and health services. Depending on how this process occurs, older adults can become dependent, resulting in the development of global syndromes, such as the frailty syndrome^(2)^.

The aging process is closely related to the frailty syndrome. It can be understood as a result of the gradual decline of physiological functions, emphasizing the decrease in the immune, muscular and neuroendocrine systems. The frailty syndrome is a multicausal condition, linked to the geriatric syndrome, characterized by low capacity for homeostasis and reduced muscle strength, resulting in a greater propensity for falls, bone fragility, malnutrition, among other adverse outcomes^(2-3)^.

The loss of independence of older adults is linked to the decrease in functional and cognitive capacity, making them need help to carry out basic daily activities. This greater dependence is often met by family members, but some families cannot deal with all the required needs, especially when they are specific to a pathology or procedure. Thus, sometimes, hospitalization in a hospital institution is seen positively by the family^(4)^.

However, negative aspects of hospitalizations must be considered. Isolation, adapting to routine, precarious conditions in many institutions and lack of professional education can expose older adults to a greater risk of violence. Risk of violence may be related to physical and emotional vulnerability, which many find themselves, as aggression is not only physical, but also psychological, or even in the absence of care, in a way that brings harm to older adults^(4-5)^.

The act of violence, single or repeated, can be classified into psychological violence, self-neglect, negligence, financial violence, sexual violence and abandonment. Also, categories can be highlighted that take into account the environment in which they are inserted, such as institutional, domestic or symbolic abuse. Whatever the type of violence, this act violates human dignity, leaving impacts on physical and psychological health^(6-7)^.

Violence is a global problem that permeates the physical act, and can be classified as physical, psychological, financial, sexual, in addition to omission and abdication. Violence is an act that hurts human dignity and negatively impacts individuals’ physical and mental health. The aggressor, in most cases, takes advantage of older adults’ frailty to impose their will. It should also be noted that violence itself can be a factor that determines older adults’ frailty^(7-8)^.

Frailty is classified based on axes that generally take into account older adults’ physical, psychological and social status; therefore, the decrease in these aspects can bring older adults closer to a state considered frail. This state leads to vulnerability and, consequently, greater exposure to risk of violence, which can be seen as an aggravating factor to the frailty syndrome, as the risk or even the act has psychological consequences, which may reflect on the physical part^(2,8-9)^.

Exposure to risk of violence, combined with health problems and distance from family and friends, can negatively affect older adults’ quality of life and, consequently, intensify the frailty process. However, there is a lack of research linking risk of violence and the frailty syndrome in hospitalized older adult^(4,7)^.

Data showed that one in six older adults has already suffered some type of violence and that this rate can be even higher in older adults who are in institutions, making hospital environments an appropriate place to assess risk of violence. Several older adults, due to their vulnerability and susceptibility to the development of comorbidities and their aggravation, need hospitalization to meet their health demands, being crucial that health professionals are alert and able to assess and care for older adults beyond the pathology^(10-11)^.

Therefore, considering the scarcity of literature that jointly assesses risk of violence and frailty syndrome, the need to encourage research is highlighted so that the creation and implementation of legislation on this topic can be encouraged. Moreover, nursing attention is drawn to these conditions, which represent a substantial drop in well-being, allowing a greater chance of death. Early detection and carrying out actions related to risk of violence are of paramount importance, as they can address actions that pose a risk to older adults’ integrity.

## OBJECTIVES

To analyze the association between risk of violence and frailty syndrome among hospitalized older adults.

## METHODS

### Ethical aspects

This research was submitted and approved by the Research Ethics Committee of the Hospital *Universitário Lauro Wanderley* (HULW/UFPB) and the *Hospital Universitário Alcides Carneiro* (HUAC/ UFCG). Older adults were invited to participate and received guidance, consenting by signing the Informed Consent Form (ICF). All recommendations and ethical principles provided for in research involving human beings were respected and followed in accordance with Resolution 466/2012, established by the Brazilian National Health Council.

### Study design, period, and location

This is a multicenter study, with a quantitative, analytical, crosssectional approach, guided by the STrengthening the Reporting of OBservational studies in Epidemiology (STROBE). The research was carried out at HULW/UFPB and HUAC/UFCG, both in Paraíba. Data collection took place from July to September 2019 at HULW/ UFPB and October 2019 to February 2020 at HUAC/UFCG.

The collection was carried out in some sectors of the hospitals, namely: at the HULW/UFPB, geriatrics and surgical clinic, medical clinic and contagious and parasitic disease unit (CPD); at HUAC/ UFCG, Wing A Surgical, Wing B Pneumo, Wing C Women’s Clinic, Wing D Men’s Clinic. The Intensive Care Unit was excluded due to the complexity of care.

### Population and sample; inclusion and exclusion criteria

The sample calculation equation for finite population study determined the sample size. The number of participants was based on the sample calculation, which took into account the number of older adults admitted and assisted in the same period of 2018. A confidence level of 95% and a frequency of violence in older adults of 60% were adopted, based on the prevalence of elder abuse. However, because the research included older adults at risk of violence, this prevalence was estimated at 60%^(9)^.

Thus, the sample consisted of 285, of which 193 were from the HULW/UFPB and 120 were from the HUAC/UFCG. Taking into account possible losses, 10% was added, totaling 323 older adults in the final sample. Non-probabilistic sampling was used, by quota, with older adults proportionally distributed among the sectors included.

Individuals aged 60 years or older assisted by the aforementioned hospital services were included, not taking into account the cause of hospitalization. Those in the terminal stage (n=23), severe difficulty in communicating (n=12), clinical conditions that prevented participation (n=10) or severe cognitive impairment (n=1) were excluded, the latter being assessed by the researcher or informed by industry professionals.

### Study protocol

Sociodemographic data were collected through a clipping of the Brazil Old Age Schedule (BOAS), in which literacy, marital status, age, wage income, sex and currently working were selected^(12)^. Violence risk was assessed by the Hwalek-Sengstock Elder Abuse Screening Test (H-S/EAST)^(13)^, and the frailty syndrome, by the Edmonton Frail Scale (EFS)^(14)^.

The H-S/EAST is an American instrument cross-culturally adapted to Brazilian Portuguese. With 15 items, the instrument assigns one point to affirmative answers, with the exception of items 1,6,12 and 14, which score as negative. The instrument assesses the risk of psychological and physical violence, violation of personal rights, isolation or financial violence. In assessing the instrument’s total score, a cut-off point of three was adopted, classifying a score above three as a risk of suffering violence.

The EFS proposes to analyze individuals’ frailty level. The classification takes place according to the pre-established score, with the levels denominated as without frailty and with frailty. Scores from zero to six are classified as without frailty; greater than six classify as frail.

Data referring to sociodemographic status (sex, currently working, age, education, marital status and income) and frailty were adopted as independent variables, whereas the dependent variable studied was risk of violence.

### Analysis of results, and statistics

Data were digitized and analyzed using the Statistical Package for the Social Sciences (SPSS), version 21.0. For data treatment, absolute and relative frequencies were performed for description, and Pearson’s chi-square test, Fisher’s exact test, Spearman’s correlation coefficient, multiple logistic regression model for inferential analysis. For cases with cells with a frequency of less than 5 (greater than 20%), Fisher’s exact test was used.

The Kolmogorov-Smirnov test for normality determined that the variables did not tend to a normal distribution, so the nonparametric test was used. The significance level of 5% (p<0.05) was adopted for all analyzes.

For the multiple regression model, variables that presented a *p* value <0.2 in the analysis of the association of frailty domains with risk of violence were included. The method used in the model was the hierarchical one, in which the largest *p* value that was not within the significance level was removed until all were in agreement with that level. The criterion to remain in the final model was to present a *p* value <0.05.

## RESULTS

The study had a sample composition of 323 older adults with a minimum age of 60 and a maximum of 93 years, with a mean age of 70.82 years and a standard deviation of 7.66. As for age, it was analyzed in a dichotomized way, taking into account the median; thus, it was observed that 52.6% (n=170) older adults were between 60 and 70 years old.

Regarding sex, there was a prevalence among women (n=196; 60.7%), who could read and write (n=219;67.8%), and 63.4% (n=204) studied more than 3 years old. It is also noted that 51.7% (n=167) were in a relationship, 78.3% (n=253) did not perform paid work and 57.9% (n=187) earned on average one minimum wage.

Table 1 demonstrates the association between risk of violence and sociodemographic variables, in which it is observed that there was a significant association between risk of violence and being female (p=0.004) and prevalence of risk among older adults (n=99; 64.7%), who cannot read and write (n=71; 68.9%), without relationship (n=104; 67.1%), without paid activity (n=164; 65.1%) and with income above 1 minimum wage (n=88; 65.2%).

**Table 1:** Association of risk of violence and sociodemographic data, João Pessoa/Campina Grande, Paraíba, Brazil, 2019-2020 (N=323)

Variables	Risk of violence With Without n (%) n (%)		*p* value
Sex Male Female	67 (53.2%) 135 (68.9%)	59 (46.8%) 61 (31.1%)	**0.004**
Age Up to 70 years Over 70 years	103 (60.9%) 99 (64.7%)	66 (39.1%) 54 (35.3%)	0.486
Literate Yes No	131 (59.8%) 71 (68.9%)	88 (40.2%) 32 (31.1)	0.115
Years of study Below or equal to 3	78 (67.2)	38 (32.8)	0.216
Over 3	71 (68.9)	32 (31.1)	
Marital status No relationship With relationship	104 (67.1) 97 (58.4)	51 (32.9) 69 (41.6)	0.109
Paid activity Yes No	38 (54.3) 164 (65.1)	32 (45.7) 88 (34.9)	0.098
Income Up to 1 minimum wage Above 1 minimum wage	114 (61.0) 88 (65.2)	73 (39.0) 47 (34.8)	0.439

* *Pearson’s chi-square test.*

The association between sociodemographic variables and risk of violence among older adults was assessed. The H-S/EAST score determined the “at risk” and “not risk” categories for violence. Table 1 demonstrates a statistically significant association between risk of violence and being female (p=0.004).

Table 2 expresses the association between risk of violence and frailty domains through EFS, in which it is possible to verify the statistical significance of risk with general health status (p=0.038), functional independence (p=0.024), social support (p=0.001), forgetting to take medication (p<0.001), mood (p<0.001) and continence (p=0.040). With regard to frailty syndrome, there was also statistical significance between risk of violence and the frailty classification (p<0.001).

**Table 2:** Association of risk of violence and dimensions of participants’ Edmonton Frail Scale, João Pessoa/Campina Grande, Paraíba, Brazil, 2019-2020

EFS domains	Risk of violence With Without n (%) n (%)		*p* value^*^
Cognition (CDT) Approved	24 (49.0)	25 (51.0)	
Disapproved with minimum errors	27 (42.9)	36 (57.1)	0.066
Disapproved with significant errors	69 (32.9)	141 (67.1)	
General health status (self-reported) Good	105 (39.8)	159 (60.2)	0.038
Bad	15 (26.3)	42 (73.7)	
Functional independence No	89 (41.2)	127 (58.8)	0.024
Yes	31 (29.2)	75 (70.8)	
Social support (Do you have someone to count on when you need help?) Always	112 (41.5)	158 (58.5)	
Sometimes	7 (16.3)	36 (83.7)	0.001^**^
Never	1 (11.1)	202 (62.7)	
Medication Do you use five or more medications? No	124 (60.8)	80 (39.2)	0.203^*^
Yes	78 (66.1)	40 (33.9)	
Do you sometimes forget to take your medication? No	86 (53.1)	76 (43.9)	**<0.001**
Yes	116 (72.5)	44 (27.5)	
Nutrition (weight loss) No	54 (37.5)	90 (62.5)	0.515^*^
Yes	66 (37.1)	112 (62.9)	
Mood (feeling sad or depressed) No	96 (53.3)	84 (46.7)	**<0.001**
Yes	24 (16.9)	118 (83.1)	
Continence (loss of urine) Yes	44 (30.6)	100 (69.4)	0.017^*^
No	76 (42.7)	102 (57.3)	
Functional performance 0 to 10 seconds	44 (41.5)	62 (58.5)	
11 to 20 seconds	44 (43.1)	58 (56.9)	0.040^*^
More than 20 seconds	32 (28.1)	82 (71.9)	
Frailty syndrome Without frailty	68 (54.0)	58 (46.0)	**<0.001**
With frailty	52 (26.5)	144 (73.5)	

EFS - Edmonton Frail Scale; H-S/EAST - Hwalek-Sengstock Elder Abuse Screening Test;

* Pearson’s chi-square test;

** Fisher’s exact test; CDT - Clock Drawing Test.

**Table 3:** Multiple regression model of risk association for violence and participants’ Edmonton Frail Scale dimensions, João Pessoa/Campina Grande, Paraíba, Brazil, 2019-2020

EFS domain	OR	Risk of violence CI	*p* value^*^
Social support Always	-	-	-
Sometimes	-	-	-
Never	2.89	1.37 – 6.08	0.005
Mood No	-	-	-
Yes	5.05	2.93 – 8.72	0.000
Medications No	-	-	-
Yes	2.24	1.35 – 3.73	0.002
Functional performance 0 to 10 seconds	-	-	-
11 to 20 seconds	-	-	-
More than 20 seconds	1.40	1.02 – 1.91	0.035

EFS - Edmonton Frail Scale; H-S/EAST - Hwalek-Sengstock Elder Abuse Screening Test; R^2^=0.26; OR - Odds Ratio; CI - Confidence Interval;

* Test significance.

**Table 4:** Correlation of frailty with risk of violence, João Pessoa/Campina Grande, Paraíba, Brazil, 2019-2020

Variables	H-S/EAST score Correlation coefficient	*p* value^*^
Escore da EFS	0.350	<0.001

EFS - Edmonton Frail Scale; H-S/EAST - Hwalek-Sengstock Elder Abuse Screening Test;

* Spearman’s correlation test.

All variables that had a *p* value <0.2 within the EFS domains were included in the regression model, such as cognition, general health status, functional independence, social support, forgetting to take medication, mood, continence, functional performance and frailty syndrome. However, only the social support, mood, medication and functional performance facets remained in the final model.

It is possible to infer that older adults without social support are 2.89 more likely to be at risk of violence and 5.05 more likely among older adults who feel depressed and sad. Forgetting to use medication has 2.24 more possibilities of risk of violence and those with slower functional performance 1.40 times.

The correlation analysis between the total EFS and H-S/EAST score demonstrates a positive correlation between the total EFS and H-S/EAST score, allowing one to say that one score increases as the other also increases. This correlation was statistically significant (*p* value <0.001).

## DISCUSSION

The lack of studies in the literature that relate risk of violence and frailty among older adults made it difficult to form a theoretical basis that would allow data comparison. Thus, although this study is multicenter, the scarcity of studies on the same subject made it difficult to generalize the results.

In this study, the prevalence of risk of violence in the female population showed a significant association (p=0.004), which converges with the literature^(15-16)^ that justifies this condition through greater longevity among women^(17-18)^. Living longer also represents a greater exposure to the consequences of aging, represented by functional decline and, thus, greater dependence on daily activities, which make them more vulnerable to suffering violence^(19-20)^.

With regard to age group and education, there was a prevalence of risk of violence among older adults and those with more than 3 years of study, which can be justified, respectively, by decline in motor and cognitive functions, increasing frailty and making older adults more dependent and vulnerable to the action of potential aggressors^(21-23)^. Through literacy and health education, older adults now have access to information about their rights, which makes complaints of violence more recurrent^(24)^.

Data related to marital status and work activity demonstrate that there is a predominance of risk of violence in older adults without a relationship and without working in paid work, where the lack of a partner and social relationships can bring a feeling of isolation, which impacts on individuals’ mental health, and older adults who do not remain active are more frail and vulnerable to risk of violence^(21,25-26)^. In terms of income, there was a greater risk among those who received more than 1 salary, which leads us to one of the most frequent types of violence, financial violence, where aggressors tend to abuse older adults with higher incomes^(8,23)^.

With regard to this, there was a significant association (<0.001) between the variables risk of violence and the frailty syndrome, and it was observed that older adults considered to be affected by frailty are more prone to violence. It is worth mentioning that the EFS assesses the level of frailty when considering the relationship between nine domains distributed among biological, psychological and social factors. Thus, the more affected individuals are, the more vulnerable they will be and, in turn, the more prone to violence^(2,27)^.

In the relationship between frailty and risk of violence, vulnerability can be recognized as a common point between frail individuals and those prone to violence. As the level of vulnerability of older adults increases, the greater the possibility of being affected by different types of violence, especially in health institutions. Frailty is a syndrome characterized by gradual and cumulative effects on several systems^(28-29)^.

The aging process impacts functional aspects such as a decrease in muscle mass, affecting the level of activity and, subsequently, functional independence or the progressive loss of cognition. However, these characteristics may be consequences of the influence of external factors, such as negligence, which can worsen the general health status, or social isolation, considering the lack of interaction or rejection by family and friends. Such aspects can increase vulnerability and, consequently, the probability of these older adults being victims of violence^(3,29)^.

Risk of violence was associated with the facets of frailty, such as low social support, depressed mood, forgetting to take medication and poor functional performance. In this bias, we can see that older adults who isolate themselves or are isolated, are depressed, who use controlled medications and are compromised in carrying out their daily activities are more vulnerable, since these aspects negatively influence their physical and mental health, thus increasing their frailty and likelihood of suffering some type of violence^(29-31)^.

Low social support, i.e., the lack of anyone to talk to or solve problems with, which are essential aspects to conquer and maintain a support network that contributes positively to psychological and social well-being, predisposes older adults to isolation and, consequently, frailty, which may also be due to the violence suffered. From this scenario, older adults become more susceptible to developing feelings of anguish and sadness, which in turn are included in factors that increase vulnerability and the risk of suffering violence^(23,30,32-33)^.

In relation to forgetting to take medications, a trend towards memory loss can be seen with advancing age, causing older adults who do not take medications correctly to further compromise their health to the detriment of aggravation of diseases which are generally affected. This aspect has a direct impact on decreased functional performance, favoring the development of dependence in older adults when carrying out their daily activities. Studies show that the greater the dependence on older adults, the greater the development of frailty and, consequently, risk of violence or violence itself^(29,34-36)^.

The relationship between the risk variables for violence and the frailty syndrome was also analyzed, which showed a statistically significant correlation. Despite the correlation being considered low, it is feasible to reflect the implications of this result. The data show that there is a dependency between the variables, i.e., as the level of frailty increases, risk of violence also increases, in the same way it happens if we consider the otherwise.

The study findings demonstrate that frailty syndrome is related to risk of violence. Based on this perspective, it is understood that frailty in older adults can be a consequence of quality of life as well as the socioeconomic context in which they are inserted. Therefore, this study highlights the need for interventions, especially in Primary Care, in order to anticipate factors that determine frailty, establishment of well-being. Also, the importance of observing signs of violence or risk is highlighted, considering the correlation between frailty and risk of violence.

### Study limitations

The few options of instruments validated and adapted to the reality of different types of elder abuse in Brazil are highlighted, in addition to the scarcity of literature that addresses the performance of forensic nursing on this theme.

### Contributions to nursing, health, or public policies

This research, through its results, contributes to strengthening a theoretical basis on abuse against older adults and favors the development of preventive action in care. Moreover, nursing professionals can, through an integral approach to the individual, act on the cognitive, functional and social domains to recognize, anticipate or break situations of violence. Thus, this study encourages forensic nursing research and represents subsidies for evidence-based care.

## CONCLUSIONS

The results found in this research showed an association between variables risk of violence and frailty syndrome, especially among older adults who are more functionally and socially dependent. Variables are directly proportional to risk of violence, i.e., the more frail the individuals, the greater the possibility of situations of violence occurring. Variables are directly proportional to risk of violence, i.e., the more frail the older adults, the greater the risk of suffering violence. In this regard, it is perceived that the more vulnerable older adults are, characterized, for example, by the aspects of being female, being older, presenting cognitive decline, and dependent, with low social support and functional performance, the more likely to frailty, and then, to suffer some kind of violence.
